# Poly[[μ_2_-1,2-bis­(1*H*-imidazol-1-ylmeth­yl)benzene-κ^2^ *N* ^3^:*N* ^3′^](μ_2_-tereph­thalato-κ^2^ *O* ^1^:*O* ^4^)zinc(II)]

**DOI:** 10.1107/S1600536810042406

**Published:** 2010-10-23

**Authors:** Shi-Shen Zhang, Li-Jiang Chen

**Affiliations:** a Department of Applied Chemistry, Zhejiang Sci-Tech University, Hangzhou 310018, People’s Republic of China

## Abstract

In the title coordination polymer, [Zn(C_8_H_4_O_4_)(C_14_H_14_N_4_)]_*n*_, the Zn^II^ atom is coordinated by two N atoms from two 1,2-bis­(imidazol-1-ylmeth­yl)benzene ligands as well as by the two O atoms from two terephthalate ligands, confering a tetra­hedral coordination geometry. The bridging ligands generate a three-dimensional structure.

## Related literature

For related structures, see: Fan *et al.* (2005 ▶, 2006 ▶); Liu *et al.* (2007 ▶, 2008*a* ▶,*b* ▶, 2009 ▶); Yang *et al.* (2008 ▶). 
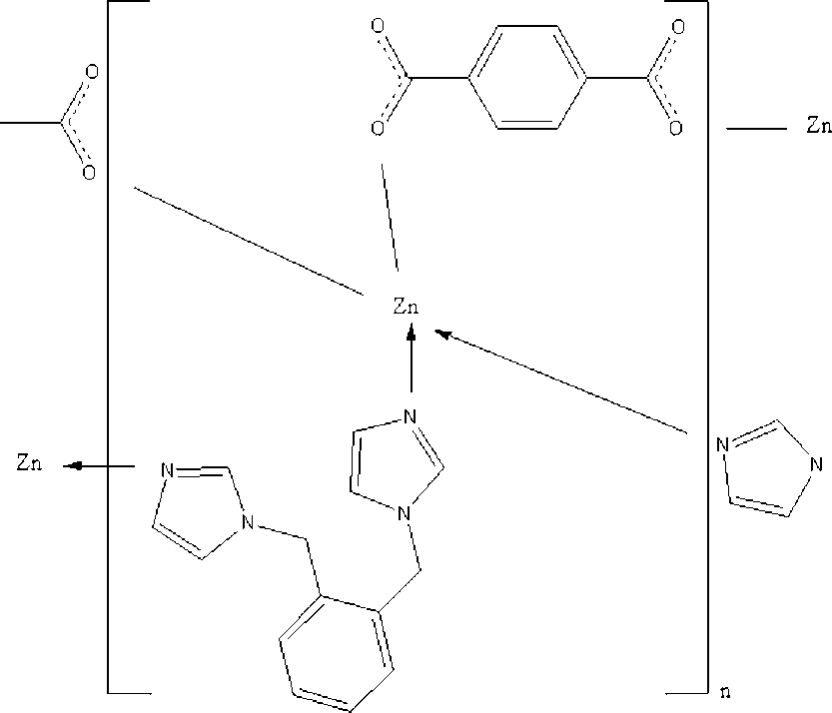


## Experimental

### 

#### Crystal data

[Zn(C_8_H_4_O_4_)(C_14_H_14_N_4_)]
*M* *_r_* = 467.79
Triclinic, 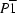

*a* = 10.132 (3) Å
*b* = 10.179 (3) Å
*c* = 11.169 (3) Å
α = 99.073 (4)°
β = 102.748 (4)°
γ = 112.974 (4)°
*V* = 995.5 (5) Å^3^
*Z* = 2
Mo *K*α radiation
μ = 1.27 mm^−1^
*T* = 291 K
0.20 × 0.16 × 0.10 mm

#### Data collection

Bruker SMART area-detector diffractometer
Absorption correction: multi-scan (*SADABS*; Sheldrick, 1996 ▶) *T*_min_ = 0.785, *T*_max_ = 0.883
7505 measured reflections
3674 independent reflections
3140 reflections with *I* > 2σ(*I*)
*R*_int_ = 0.027

#### Refinement

*R*[*F* ^2^ > 2σ(*F* ^2^)] = 0.042
*wR*(*F* ^2^) = 0.097
*S* = 1.05
3674 reflections
298 parameters
H-atom parameters constrained
Δρ_max_ = 0.60 e Å^−3^
Δρ_min_ = −0.23 e Å^−3^

### 

Data collection: *SMART* (Bruker, 1998 ▶); cell refinement: *SAINT* (Bruker, 1998 ▶); data reduction: *SAINT*; program(s) used to solve structure: *SHELXS97* (Sheldrick, 2008 ▶); program(s) used to refine structure: *SHELXL97* (Sheldrick, 2008 ▶); molecular graphics: *SHELXTL* (Sheldrick, 2008 ▶); software used to prepare material for publication: *SHELXTL*.

## Supplementary Material

Crystal structure: contains datablocks I, global. DOI: 10.1107/S1600536810042406/ng5050sup1.cif

Structure factors: contains datablocks I. DOI: 10.1107/S1600536810042406/ng5050Isup2.hkl

Additional supplementary materials:  crystallographic information; 3D view; checkCIF report

## Figures and Tables

**Fig. 1.**
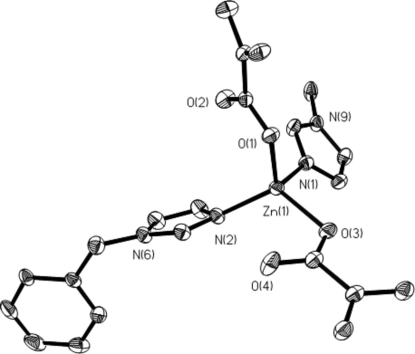
The asymmetric unit of the title compound showing 30% probability ellipsoids.

**Fig. 2.**
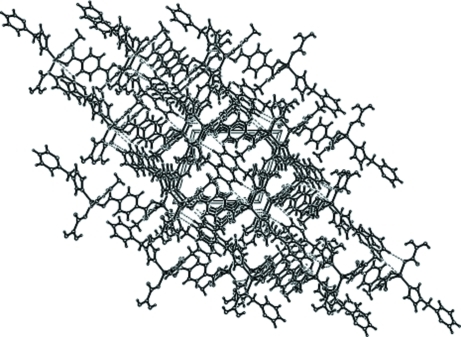
The crystal packing of the title compound

## supplementary crystallographic information

### Comment

Imidazol and its derivatives have been achieving rapidly increasing attention not only for their potential application as functional materials, but also for their intriguing variety of architectures and topologies. 1,2-bis(imidazole-1-ylmethyl)-benzene, as one kind of those ligand, has usually been used to construct a great variety of structurally interesting entities, such as one-dimensional chain, square grid, 2-fold interpenetrated, 3-fold interpenetrated network.

The asymmetric unit of the title compound (I) is illustrated in Fig. 1. Single-crystal X-ray diffraction shows that the asymmetric unit contains one Zn crystallographically nonequivalent atom.the Zn^II^ atom is coordinated by two N atoms from two 1,2-bis(imidazole-1-ylmethyl)-benzene ligands, as well as by the two O atoms from two terephthalic acid ligands to confer a distorted tetrahedral coordination at the metal centre. The two Zn atoms coordinated by two N atoms to form a layer.  The layer three-dimensional structure is stabilized by intermolecular π-π stacking interaction and hydrogen bond.

### Experimental

A mixture of ZnSO_4_ (0.032 g, 0.2 nmol), 1,2-bis(imidazole-1-ylmethyl)-benzene(0.024 g, 0.1 nmol) and Terephthalic acid (0.016 g, 0.1 nmol) in mole ratio of 1:1:1 in water(6 ml) was sealed in 15 ml Teflon-lined reactor and heated to 180°C for 24 h and then cooled to room temperature at a rate of 5°C/h. the yellow block crystal was obtianed in the yield of 20%.

### Refinement

H atoms were positioned geometrically and refined using a riding model, with C—H = 0.93 Å(aromatic) or 0.97 Å(aliphatic), and with *U*_iso_(H) = 1.2*U*_eq_(C,*N*).

### Crystal data

**Table:**

[Zn(C_8_H_4_O_4_)(C_14_H_14_N_4_)]	*Z* = 2
*M**_r_* = 467.79	*F*(000) = 480
Triclinic, *P*1	*D*_x_ = 1.561 Mg m^−^^3^
Hall symbol: -P 1	Mo *K*α radiation, λ = 0.71073 Å
*a* = 10.132 (3) Å	Cell parameters from 1793 reflections
*b* = 10.179 (3) Å	θ = 1.9–25.5°
*c* = 11.169 (3) Å	µ = 1.27 mm^−^^1^
α = 99.073 (4)°	*T* = 291 K
β = 102.748 (4)°	Block, yellow
γ = 112.974 (4)°	0.20 × 0.16 × 0.10 mm
*V* = 995.5 (5) Å^3^	

### Data collection

**Table:**

Bruker SMART area-detector diffractometer	3674 independent reflections
Radiation source: fine-focus sealed tube	3140 reflections with *I* > 2σ(*I*)
graphite	*R*_int_ = 0.027
φ and ω scans	θ_max_ = 25.5°, θ_min_ = 1.9°
Absorption correction: multi-scan (*SADABS*; Sheldrick, 1996)	*h* = −12→12
*T*_min_ = 0.785, *T*_max_ = 0.883	*k* = −12→12
7505 measured reflections	*l* = −13→13

### Refinement

**Table:**

Refinement on *F*^2^	Primary atom site location: structure-invariant direct methods
Least-squares matrix: full	Secondary atom site location: difference Fourier map
*R*[*F*^2^ > 2σ(*F*^2^)] = 0.042	Hydrogen site location: inferred from neighbouring sites
*wR*(*F*^2^) = 0.097	H-atom parameters constrained
*S* = 1.05	*w* = 1/[σ^2^(*F*_o_^2^) + (0.0482*P*)^2^ + 0.0138*P*] where *P* = (*F*_o_^2^ + 2*F*_c_^2^)/3
3674 reflections	(Δ/σ)_max_ < 0.001
298 parameters	Δρ_max_ = 0.60 e Å^−^^3^
0 restraints	Δρ_min_ = −0.23 e Å^−^^3^

### Special details

**Table:**

Geometry. All e.s.d.'s (except the e.s.d. in the dihedral angle between two l.s. planes) are estimated using the full covariance matrix. The cell e.s.d.'s are taken into account individually in the estimation of e.s.d.'s in distances, angles and torsion angles; correlations between e.s.d.'s in cell parameters are only used when they are defined by crystal symmetry. An approximate (isotropic) treatment of cell e.s.d.'s is used for estimating e.s.d.'s involving l.s. planes.
Refinement. Refinement of *F*^2^ against ALL reflections. The weighted *R*-factor *wR* and goodness of fit *S* are based on *F*^2^, conventional *R*-factors *R* are based on *F*, with *F* set to zero for negative *F*^2^. The threshold expression of *F*^2^ > σ(*F*^2^) is used only for calculating *R*-factors(gt) *etc*. and is not relevant to the choice of reflections for refinement. *R*-factors based on *F*^2^ are statistically about twice as large as those based on *F*, and *R*- factors based on ALL data will be even larger.

### Fractional atomic coordinates and isotropic or equivalent isotropic displacement parameters (Å^2^)

**Table:**

	*x*	*y*	*z*	*U*_iso_*/*U*_eq_	
Zn1	0.72744 (4)	0.03060 (3)	−0.12258 (3)	0.03123 (13)	
O1	0.8199 (2)	−0.0226 (2)	−0.24812 (18)	0.0371 (5)	
O2	0.9037 (2)	0.2104 (2)	−0.2633 (2)	0.0458 (6)	
O3	0.5956 (2)	−0.1577 (2)	−0.0977 (2)	0.0459 (6)	
O4	0.8048 (3)	−0.1373 (2)	0.0314 (3)	0.0560 (6)	
N1	0.5722 (3)	0.0954 (3)	−0.2066 (2)	0.0348 (6)	
N2	0.8687 (3)	0.1981 (2)	0.0314 (2)	0.0337 (6)	
N6	1.0855 (3)	0.3509 (3)	0.1741 (2)	0.0360 (6)	
N9	0.4735 (3)	0.2181 (3)	−0.3094 (2)	0.0376 (6)	
C1	0.9907 (4)	0.1328 (4)	−0.4772 (3)	0.0455 (8)	
H1	0.9858	0.2230	−0.4625	0.073 (12)*	
C4	0.8876 (3)	0.0814 (3)	−0.2957 (3)	0.0314 (6)	
C6	0.9462 (3)	0.0392 (3)	−0.4010 (3)	0.0319 (7)	
C9	0.9965 (3)	0.2102 (3)	0.1035 (3)	0.0360 (7)	
H9	1.0222	0.1322	0.1055	0.045 (9)*	
C10	0.6685 (4)	−0.2048 (3)	−0.0265 (3)	0.0382 (7)	
C11	0.8774 (4)	0.3382 (3)	0.0577 (3)	0.0446 (8)	
H11	0.8025	0.3636	0.0203	0.052 (10)*	
C13	1.2799 (3)	0.5047 (3)	0.3833 (3)	0.0356 (7)	
C18	1.4214 (3)	0.6254 (3)	0.4431 (3)	0.0394 (7)	
C20	0.5801 (3)	−0.3584 (3)	−0.0123 (3)	0.0382 (7)	
C22	1.3428 (5)	0.6714 (4)	0.6276 (3)	0.0633 (11)	
H22	1.3647	0.7271	0.7097	0.067 (11)*	
C23	1.1735 (4)	0.4697 (3)	0.4470 (3)	0.0420 (8)	
H23	1.0795	0.3895	0.4065	0.035 (8)*	
C26	0.5945 (3)	0.1929 (3)	−0.2730 (3)	0.0389 (7)	
H26	0.6833	0.2390	−0.2922	0.030 (7)*	
C27	0.4285 (3)	0.0549 (3)	−0.2007 (3)	0.0407 (7)	
H27	0.3809	−0.0136	−0.1596	0.047 (9)*	
C28	0.6533 (4)	−0.4224 (3)	0.0621 (3)	0.0443 (8)	
H28	0.7563	−0.3708	0.1043	0.050 (10)*	
C31	0.9565 (4)	−0.0932 (3)	−0.4253 (3)	0.0436 (8)	
H31	0.9278	−0.1572	−0.3750	0.063 (11)*	
C32	0.4269 (4)	−0.4382 (3)	−0.0747 (3)	0.0442 (8)	
H32	0.3775	−0.3967	−0.1252	0.058 (10)*	
C33	1.2432 (3)	0.4081 (4)	0.2513 (3)	0.0441 (8)	
H33A	1.2660	0.3252	0.2594	0.063 (11)*	
H33B	1.3073	0.4652	0.2071	0.042 (9)*	
C37	1.0093 (4)	0.4329 (3)	0.1455 (3)	0.0443 (8)	
H37	1.0422	0.5339	0.1795	0.038 (8)*	
C38	1.2048 (4)	0.5524 (4)	0.5688 (3)	0.0547 (9)	
H38	1.1325	0.5272	0.6106	0.040 (9)*	
C39	1.4495 (4)	0.7073 (4)	0.5666 (3)	0.0562 (10)	
H39	1.5423	0.7885	0.6075	0.065 (11)*	
C45	0.4583 (3)	0.3249 (4)	−0.3803 (3)	0.0490 (9)	
H45A	0.3600	0.2789	−0.4446	0.045 (9)*	
H45B	0.4650	0.4104	−0.3222	0.045 (9)*	
C46	0.3671 (4)	0.1301 (3)	−0.2636 (3)	0.0431 (8)	
H46	0.2710	0.1233	−0.2735	0.052 (10)*	

### Atomic displacement parameters (Å^2^)

**Table:**

	*U*^11^	*U*^22^	*U*^33^	*U*^12^	*U*^13^	*U*^23^
Zn1	0.0359 (2)	0.0264 (2)	0.0302 (2)	0.01204 (16)	0.01047 (15)	0.00882 (14)
O1	0.0418 (12)	0.0331 (11)	0.0389 (12)	0.0145 (10)	0.0196 (10)	0.0115 (9)
O2	0.0562 (14)	0.0343 (12)	0.0543 (14)	0.0227 (11)	0.0270 (12)	0.0103 (10)
O3	0.0583 (15)	0.0309 (12)	0.0426 (13)	0.0103 (11)	0.0167 (11)	0.0174 (10)
O4	0.0445 (14)	0.0400 (14)	0.0877 (18)	0.0156 (11)	0.0265 (13)	0.0285 (13)
N1	0.0354 (14)	0.0328 (14)	0.0356 (14)	0.0136 (11)	0.0101 (11)	0.0127 (11)
N2	0.0378 (14)	0.0285 (13)	0.0316 (13)	0.0149 (11)	0.0062 (11)	0.0050 (11)
N6	0.0413 (15)	0.0303 (14)	0.0310 (13)	0.0149 (12)	0.0072 (12)	0.0021 (11)
N9	0.0292 (13)	0.0356 (14)	0.0442 (15)	0.0129 (11)	0.0050 (12)	0.0132 (12)
C1	0.065 (2)	0.0356 (18)	0.055 (2)	0.0290 (17)	0.0345 (18)	0.0205 (16)
C4	0.0277 (15)	0.0310 (16)	0.0322 (16)	0.0114 (13)	0.0076 (13)	0.0064 (13)
C6	0.0295 (15)	0.0303 (16)	0.0355 (16)	0.0108 (13)	0.0131 (13)	0.0099 (13)
C9	0.0442 (18)	0.0294 (16)	0.0335 (16)	0.0177 (14)	0.0108 (14)	0.0041 (13)
C10	0.047 (2)	0.0300 (17)	0.0406 (18)	0.0159 (15)	0.0230 (16)	0.0068 (14)
C11	0.054 (2)	0.0357 (18)	0.0436 (19)	0.0276 (17)	0.0028 (16)	0.0059 (15)
C13	0.0429 (18)	0.0308 (16)	0.0307 (16)	0.0170 (14)	0.0045 (14)	0.0095 (13)
C18	0.0387 (17)	0.0311 (16)	0.0396 (18)	0.0133 (14)	−0.0029 (14)	0.0138 (14)
C20	0.0467 (19)	0.0323 (16)	0.0418 (18)	0.0176 (15)	0.0227 (15)	0.0130 (14)
C22	0.090 (3)	0.050 (2)	0.0291 (19)	0.023 (2)	0.002 (2)	0.0006 (17)
C23	0.0468 (19)	0.0297 (17)	0.0389 (18)	0.0094 (15)	0.0094 (15)	0.0065 (14)
C26	0.0306 (17)	0.0350 (17)	0.052 (2)	0.0131 (14)	0.0115 (15)	0.0189 (15)
C27	0.0395 (18)	0.0410 (18)	0.0437 (18)	0.0156 (15)	0.0193 (15)	0.0142 (15)
C28	0.043 (2)	0.0381 (18)	0.053 (2)	0.0153 (16)	0.0167 (17)	0.0197 (16)
C31	0.059 (2)	0.0366 (18)	0.051 (2)	0.0241 (16)	0.0316 (17)	0.0223 (16)
C32	0.047 (2)	0.0376 (18)	0.053 (2)	0.0205 (16)	0.0159 (17)	0.0215 (16)
C33	0.0415 (19)	0.0430 (19)	0.0423 (19)	0.0166 (16)	0.0126 (16)	0.0033 (15)
C37	0.057 (2)	0.0293 (18)	0.0427 (18)	0.0209 (16)	0.0070 (17)	0.0055 (14)
C38	0.077 (3)	0.053 (2)	0.0397 (19)	0.030 (2)	0.023 (2)	0.0154 (17)
C39	0.059 (2)	0.040 (2)	0.040 (2)	0.0102 (18)	−0.0127 (18)	0.0063 (16)
C45	0.0325 (18)	0.040 (2)	0.064 (2)	0.0126 (15)	−0.0018 (17)	0.0198 (18)
C46	0.0337 (18)	0.0428 (19)	0.053 (2)	0.0159 (15)	0.0153 (15)	0.0129 (16)

### Geometric parameters (Å, °)

**Table:**

Zn1—O3	1.967 (2)	C13—C33	1.515 (4)
Zn1—O1	1.9680 (19)	C18—C39	1.399 (5)
Zn1—N2	1.999 (2)	C18—C45^ii^	1.497 (4)
Zn1—N1	2.036 (2)	C20—C32	1.384 (4)
O1—C4	1.283 (3)	C20—C28	1.393 (4)
O2—C4	1.240 (3)	C22—C39	1.364 (5)
O3—C10	1.244 (4)	C22—C38	1.369 (5)
O4—C10	1.234 (4)	C22—H22	0.9271
N1—C26	1.310 (3)	C23—C38	1.378 (4)
N1—C27	1.370 (4)	C23—H23	0.9299
N2—C9	1.313 (4)	C26—H26	0.9298
N2—C11	1.373 (4)	C27—C46	1.347 (4)
N6—C9	1.344 (3)	C27—H27	0.9297
N6—C37	1.369 (4)	C28—C32^iii^	1.381 (4)
N6—C33	1.464 (4)	C28—H28	0.9299
N9—C26	1.339 (4)	C31—C1^i^	1.386 (4)
N9—C46	1.364 (4)	C31—H31	0.9294
N9—C45	1.478 (4)	C32—C28^iii^	1.381 (4)
C1—C31^i^	1.386 (4)	C32—H32	0.9283
C1—C6	1.387 (4)	C33—H33A	0.9696
C1—H1	0.9289	C33—H33B	0.9695
C4—C6	1.506 (4)	C37—H37	0.9299
C6—C31	1.383 (4)	C38—H38	0.9292
C9—H9	0.9291	C39—H39	0.9280
C10—C20	1.522 (4)	C45—C18^ii^	1.497 (4)
C11—C37	1.340 (4)	C45—H45A	0.9699
C11—H11	0.9294	C45—H45B	0.9696
C13—C23	1.383 (4)	C46—H46	0.9284
C13—C18	1.398 (4)		
			
O3—Zn1—O1	105.82 (9)	C28—C20—C10	120.3 (3)
O3—Zn1—N2	118.54 (9)	C39—C22—C38	120.2 (3)
O1—Zn1—N2	115.65 (9)	C39—C22—H22	119.6
O3—Zn1—N1	100.79 (10)	C38—C22—H22	120.1
O1—Zn1—N1	109.17 (9)	C38—C23—C13	120.9 (3)
N2—Zn1—N1	105.62 (10)	C38—C23—H23	119.8
C4—O1—Zn1	115.41 (18)	C13—C23—H23	119.2
C10—O3—Zn1	111.8 (2)	N1—C26—N9	111.6 (3)
C26—N1—C27	105.7 (2)	N1—C26—H26	124.3
C26—N1—Zn1	125.8 (2)	N9—C26—H26	124.2
C27—N1—Zn1	128.4 (2)	C46—C27—N1	109.2 (3)
C9—N2—C11	105.6 (2)	C46—C27—H27	125.4
C9—N2—Zn1	125.44 (19)	N1—C27—H27	125.3
C11—N2—Zn1	126.0 (2)	C32^iii^—C28—C20	120.3 (3)
C9—N6—C37	107.5 (3)	C32^iii^—C28—H28	119.8
C9—N6—C33	125.4 (3)	C20—C28—H28	120.0
C37—N6—C33	126.5 (3)	C6—C31—C1^i^	121.5 (3)
C26—N9—C46	106.8 (3)	C6—C31—H31	119.3
C26—N9—C45	127.3 (3)	C1^i^—C31—H31	119.2
C46—N9—C45	125.9 (3)	C28^iii^—C32—C20	121.0 (3)
C31^i^—C1—C6	120.5 (3)	C28^iii^—C32—H32	119.7
C31^i^—C1—H1	119.6	C20—C32—H32	119.3
C6—C1—H1	119.9	N6—C33—C13	113.4 (3)
O2—C4—O1	124.7 (3)	N6—C33—H33A	108.9
O2—C4—C6	119.0 (2)	C13—C33—H33A	108.9
O1—C4—C6	116.3 (2)	N6—C33—H33B	108.8
C31—C6—C1	118.0 (3)	C13—C33—H33B	109.0
C31—C6—C4	121.4 (3)	H33A—C33—H33B	107.7
C1—C6—C4	120.6 (3)	C11—C37—N6	105.9 (3)
N2—C9—N6	110.9 (3)	C11—C37—H37	126.8
N2—C9—H9	124.7	N6—C37—H37	127.2
N6—C9—H9	124.5	C22—C38—C23	119.7 (4)
O4—C10—O3	124.9 (3)	C22—C38—H38	120.3
O4—C10—C20	118.8 (3)	C23—C38—H38	120.0
O3—C10—C20	116.2 (3)	C22—C39—C18	121.5 (3)
C37—C11—N2	110.1 (3)	C22—C39—H39	119.3
C37—C11—H11	124.9	C18—C39—H39	119.3
N2—C11—H11	125.0	N9—C45—C18^ii^	111.3 (3)
C23—C13—C18	119.7 (3)	N9—C45—H45A	109.3
C23—C13—C33	119.4 (3)	C18^ii^—C45—H45A	109.5
C18—C13—C33	120.9 (3)	N9—C45—H45B	109.4
C13—C18—C39	118.0 (3)	C18^ii^—C45—H45B	109.4
C13—C18—C45^ii^	123.6 (3)	H45A—C45—H45B	108.0
C39—C18—C45^ii^	118.4 (3)	C27—C46—N9	106.7 (3)
C32—C20—C28	118.8 (3)	C27—C46—H46	126.5
C32—C20—C10	120.9 (3)	N9—C46—H46	126.8

Symmetry codes: (i) −*x*+2, −*y*, −*z*−1; (ii) −*x*+2, −*y*+1, −*z*; (iii) −*x*+1, −*y*−1, −*z*.
